# Role of macrophages during skeletal muscle regeneration and hypertrophy—Implications for immunomodulatory strategies

**DOI:** 10.14814/phy2.15480

**Published:** 2022-10-06

**Authors:** Clara Bernard, Aliki Zavoriti, Quentin Pucelle, Bénédicte Chazaud, Julien Gondin

**Affiliations:** ^1^ Institut NeuroMyoGène, Unité Physiopathologie et Génétique du Neurone et du Muscle Université Claude Bernard Lyon 1, CNRS UMR 5261, INSERM U1315, Université Lyon Lyon France; ^2^ Université de Versailles Saint‐Quentin‐En‐Yvelines Versailles France

## Abstract

Skeletal muscle is a plastic tissue that regenerates *ad integrum* after injury and adapts to raise mechanical loading/contractile activity by increasing its mass and/or myofiber size, a phenomenon commonly refers to as skeletal muscle hypertrophy. Both muscle regeneration and hypertrophy rely on the interactions between muscle stem cells and their neighborhood, which include inflammatory cells, and particularly macrophages. This review first summarizes the role of macrophages in muscle regeneration in various animal models of injury and in response to exercise‐induced muscle damage in humans. Then, the potential contribution of macrophages to skeletal muscle hypertrophy is discussed on the basis of both animal and human experiments. We also present a brief comparative analysis of the role of macrophages during muscle regeneration versus hypertrophy. Finally, we summarize the current knowledge on the impact of different immunomodulatory strategies, such as heat therapy, cooling, massage, nonsteroidal anti‐inflammatory drugs and resolvins, on skeletal muscle regeneration and their potential impact on muscle hypertrophy.

## Graphical abstract

Macrophage dynamics during muscle regeneration versus hypertrophy.

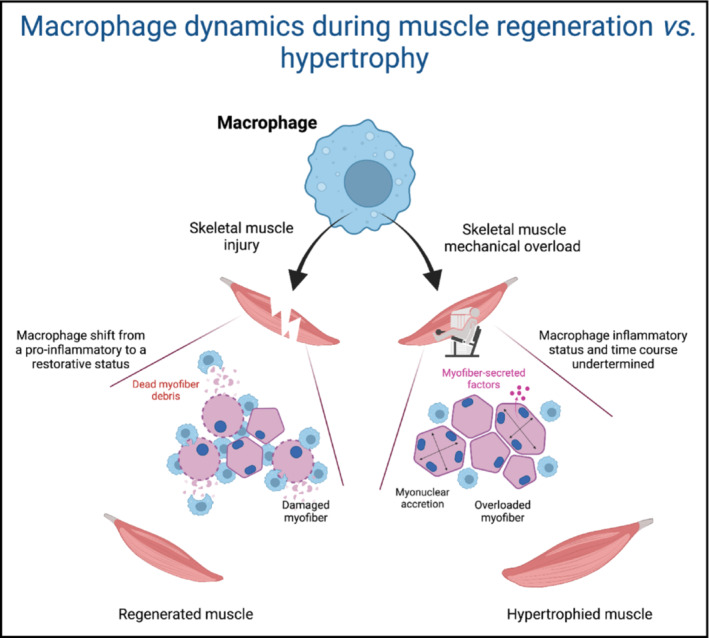

## 1 INTRODUCTION

Adult skeletal muscle is a highly plastic organ. Muscle remodels, i.e. adapts to the physical demand, such as during hypertrophy, and regenerates after a damage. The two processes involve different mechanisms (Fukada et al., 2022). Muscle remodeling mainly relies on adaptation of the myofibers while regeneration relies on muscle stem cells (aka satellite cells, MuSCs) that implement adult myogenesis. MuSCs are absolutely required for muscle regeneration (Lepper et al., 2011; Murphy et al., 2011; Sambasivan et al., 2011), but their involvement in remodeling induced by (or consecutive to) skeletal muscle hypertrophy remains a matter of debate (Egner et al., 2016; McCarthy et al., 2011). However, in both situations, MuSCs develop interactions with their neighborhood, which includes inflammatory cells, and particularly macrophages.

Macrophages are known for a long time to be involved in post‐injury skeletal muscle regeneration, where they play very important trophic roles for MuSCs and other cells in the muscle tissue. By extension, macrophages have been investigated in other conditions where muscle remodeling takes place, such as hypertrophy, but the knowledge on their biological functions is far less advanced in that context, both in animal models and human. Here we provide a brief overview on how macrophages regulate muscle regeneration through their interactions with various cell types (references to reviews for more details are given in the text), a prerequisite to understand their role in the regulation of muscle mass. Next, we address for the first time whether and to what extent macrophages are involved in muscle hypertrophy and summarize the recent advances on this topic. Then, we present a brief comparative analysis of the role of macrophages during muscle regeneration versus hypertrophy. Finally, the identification of the biological functions of macrophages in muscle regeneration and remodeling has made possible the rewiring of monitoring inflammation after an injury. Thus, the last section of this review focuses on the management of the inflammatory response through the application of widespread methods used by athletes to improve muscle recovery after an injury or to promote muscle hypertrophy in response to exercise.

## 2 MACROPHAGES AND SKELETAL MUSCLE REGENERATION

Adult skeletal muscle regenerates *ad integrum* after an injury induced by myotoxic agents (cardiotoxin, BaCl2) (Arnold et al., 2007; Hardy et al., 2016), physical muscle injury (e.g., crush or freeze injury) (Hardy et al., 2016; Takagi et al., 2011) or unaccustomed exercise (also referred to as exercise‐induced muscle damage [EIMD]) (Saclier et al., 2013). The role of macrophages in muscle regeneration is first presented in various animal models of injury and then in response to EIMD in humans.

### 2.1 Animal studies

Skeletal muscle regeneration relies on MuSC properties, that exit quiescence, expand, differentiate and fuse to form new functional myofibers. The adult myogenic program is tightly controlled by myogenic transcription factors which expression follows an intrinsic pattern (Yin et al., 2013). However, the last decade evidenced that the MuSC environment impacts on that program: well‐orchestrated environmental cues support each step of myogenesis for a harmonious and efficient regeneration. On the contrary, alteration of some properties of that environment delays or impairs the myogenic program, thus the regeneration process as a whole (Dumont et al., 2015).

After an injury, neutrophils are the first immune cells to invade the injury site where they attract monocytes and start the inflammatory response. Ly6C^pos^CCR2^pos^CX3CR1^lo^ circulating monocytes enter the damaged muscle area through the CCL2‐CCR2 axis (Contreras‐Shannon et al., 2007; Martinez et al., 2010; Warren et al., 2005) as well as via the complement protein C3a‐C3aR axis (Zhang et al., 2017). After their arrival in the damaged muscle, they become Ly6C^pos^ pro‐inflammatory macrophages. They promote the expansion of MuSCs by stimulating their proliferation through the delivery of soluble factors and metabolites (among which IL‐6, TNFα, VEGF, IGF1, IL1β; IL13, NAMPT, glutamine) (Baht et al., 2020; Lu et al., 2011; Ratnayake et al., 2021; Shang et al., 2020; Tonkin et al., 2015), they also control the number of fibro‐adipo‐progenitors (FAPs) by triggering their apoptosis through TNFα production (Lemos et al., 2015). The pro‐inflammatory phase is usually short (2–3 days) and ends with the shift towards the acquisition of an anti‐inflammatory/restorative phenotype by macrophages. A major role of macrophages is efferocytosis, which consists in the phagocytosis of dead cells and cell debris. This process induces the resolution of inflammation by the modification of the macrophage inflammatory status. Engulfment of dead myoblasts stops the secretion of TNFα and induces that of anti‐inflammatory effectors like TGF‐β (Arnold et al., 2007). The macrophage shift is characterized by metabolic changes, from a glycolytic to oxidative and glutamine metabolism (for reviews see Juban & Chazaud, 2017, 2021). Ly6C^neg^ restorative macrophages exert a series of functions while dampening the inflammatory response. They stimulate terminal myogenic differentiation and fusion of MuSCs via the secretion of factors (IGF‐1, TGF‐β, GDF3…) (Arnold et al., 2007; Lu et al., 2011; Saclier et al., 2013; Varga, Mounier, Patsalos, et al., 2016). Restorative macrophages also promote angiogenesis, they stimulate fibroblastic cells for extracellular matrix (ECM) remodeling and secrete themselves ECM components (e.g., proteoglycans, matricellular proteins and assembly proteins) (Varga, Mounier, Horvath, et al., 2016, for reviews see Panci & Chazaud, 2021; Singh & Chazaud, 2021). The kinetics of the inflammatory response is fundamental for the good orchestration of myogenesis and the surrounding supportive biological processes. Indeed, inhibition of the molecular pathways involved in the macrophage shift (including to date IGF‐1, MKP1‐p38, SRB1‐ERK, AMPK, C/EBPβ, STAT3, NFIX, BACH1) leads to a loss of acquisition of the restorative phenotype and therefore to an impairment in muscle regeneration (Baht et al., 2020; Mounier et al., 2013; Patsalos et al., 2022; Perdiguero et al., 2011; Saclier et al., 2020; Tonkin et al., 2015; Zhang et al., 2019, for review see Juban, 2021). Moreover, too early acquisition of the restorative phenotype also leads to an altered muscle regeneration (Bencze et al., 2012; Caratti et al., 2020; Cheng et al., 2008; Giannakis et al., 2019; Perdiguero et al., 2011; Rigamonti et al., 2013). In vitro analyses using human cells showed that pro‐inflammatory macrophages inhibit differentiation and fusion of MuSCs (Saclier et al., 2013), suggesting that in vivo too early appearance of restorative macrophages triggers too early myogenic differentiation/fusion, thus preventing an efficient muscle regeneration.

**FIGURE 1:**
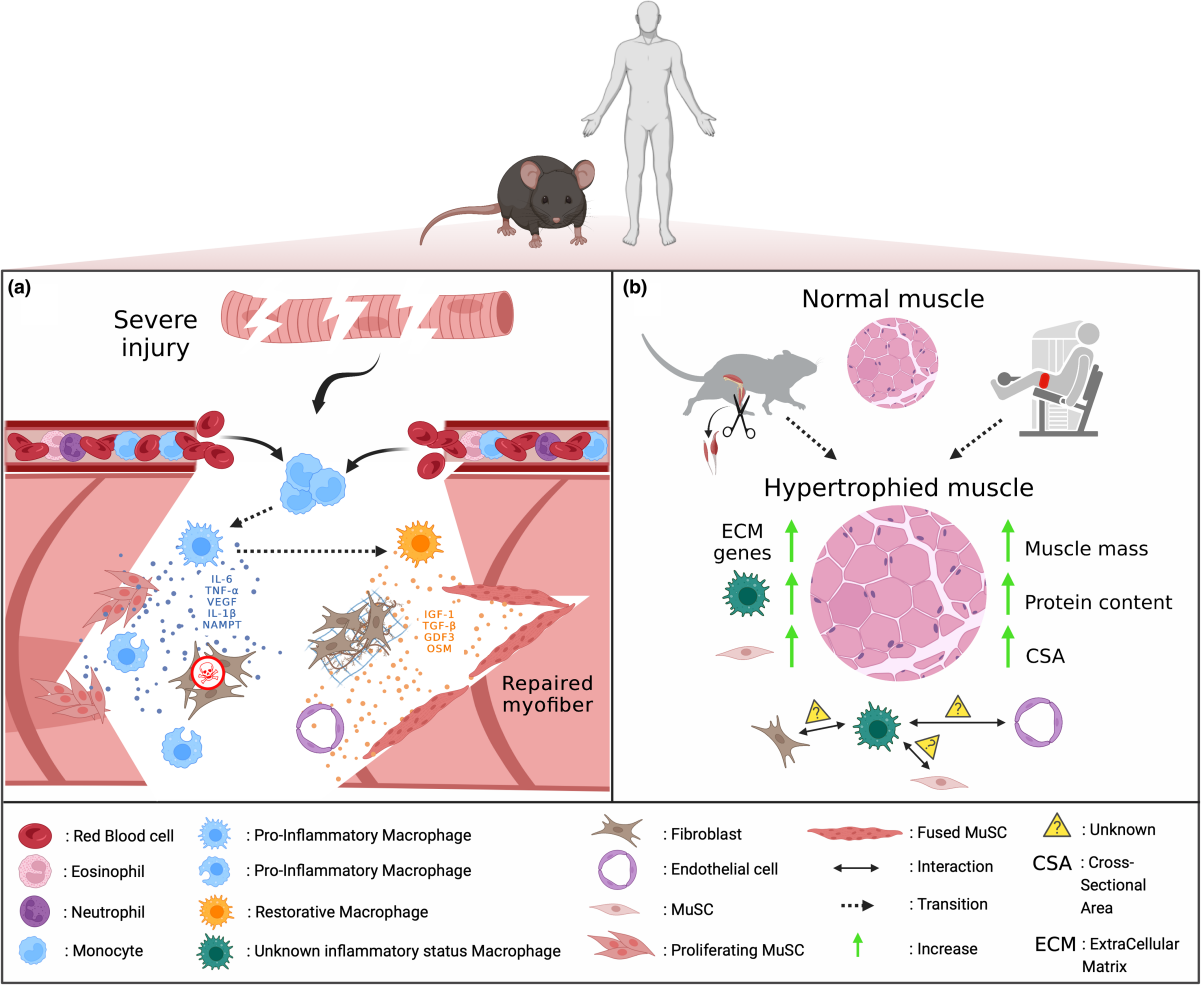
Role of macrophages during muscle regeneration and hypertrophy in animals and human. (a) after a severe injury usually induced by injection of a snake venom (e.g., cardiotoxin or notexin) into the mouse muscle, blood circulating monocytes enter the injured muscle and become pro‐inflammatory macrophages. They secrete cytokines such as IL‐6, TNF‐α, VEGF, IL‐1β and NAMPT to promote the proliferation of MuSCs and apoptosis of fibroblasts. Thanks to the phagocytosis of cell debris, pro‐inflammatory macrophages switch to become restorative macrophages and deliver other effectors such as IGF‐1, TGF‐β, GDF3 and OSM. They stimulate differentiation and fusion of MuSCs to repair the injured myofibers and promote angiogenesis and extracellular matrix remodeling. Human investigations also reported an accumulation of pro‐inflammatory macrophages in regenerating areas containing proliferating MuSCs while restorative macrophages were mainly located in areas containing differentiating MuSCs after a severe exercise‐induced muscle damage. (b) Hypertrophy can be induced experimentally by synergistic ablation of hindlimb muscles in animals or by resistance training in human, which is sometimes performed under blood flow restriction (the pneumatic cuff placed around the thigh is represented in red). These models are associated with an increase in muscle mass, myofiber cross‐sectional area, protein content, number of MuSCs, expression of genes involved in the regulation of extracellular matrix and macrophage infiltration. However, the inflammatory status of macrophages is not known in this context and how they interact with MuSCs, fibroblasts and endothelial cells to regulate changes in muscle mass has to be determined.

The various models of skeletal muscle injury, notably in the mouse, have allowed the development of an important field of investigations to understand the cellular and molecular mechanisms regulating adult skeletal muscle regeneration (Figure 1a). A limitation of these models is the extent of the damage, that covers the whole muscle (or most part of it). Thus, although very reproducible and robust, these models poorly represent injuries occurring in humans and are non‐physiological.

### 2.2 Human studies

Our knowledge on the role of macrophages in skeletal muscle regeneration mainly relies on animal models of muscle injury. In human, the accumulation of macrophages after EIMD is still a matter of debate. This is due to the unclear definition of EIMD and the related use of indirect markers, such as delayed onset muscle soreness and/or blood sampling parameters (e.g., creatine kinase), that mainly reflect symptoms of muscle damage and not necessarily its occurrence and/or magnitude. The evaluation of maximal force loss and recovery after exercise allows to overcome these limitations and is now considered as the best indirect marker of muscle damage severity (Paulsen et al., 2012; Warren et al., 1999). Indeed, a mild muscle damage, which is defined as a force decline of no more than 20% (during the first 24 h) and/or full recovery within 48 h, does not lead to macrophage infiltration in all (Bourgeois et al., 1999; Féasson et al., 2002; Malm et al., 2004) but one (Crameri et al., 2007) studies. Interestingly, a myofiber self‐repair/remodeling mechanism, independent of MuSCs, has been recently demonstrated in response to a mild injury (Roman et al., 2021), even though the lack of force measurements does not allow to determine the magnitude of muscle damage in this context. On the contrary, severe muscle damage, which corresponds to a force loss over 50% and/or recovery lasting longer than 1 week, is consistently associated with leukocyte/macrophage infiltration (Beaton et al., 2002; Child et al., 1999; Hikida et al., 1983; Jones et al., 1986; Lauritzen et al., 2009; Mackey et al., 2016; Paulsen et al., 2010; Round et al., 1987; Saclier et al., 2013), thereby illustrating a link between macrophage accumulation and the magnitude of muscle damage. In agreement with animal investigations, severe EIMD in human leads to an accumulation of pro‐inflammatory macrophages (i.e., CD68^pos^iNOS^pos^ and C68^pos^COX2^pos^ cells) in regenerating areas containing proliferating MuSCs while restorative macrophages (i.e., CD68^pos^Arg1^pos^, CD206^pos^ and CD163^pos^ cells) were mainly located in areas containing differentiating MuSCs (Saclier et al., 2013). Finally, an elegant analysis of human single myofibers after EIMD shows macrophage infiltration in both necrotic and regenerating muscle regions to remove damaged tissue and promote myogenesis, respectively (Mackey & Kjaer, 2017). These findings illustrate the key role of macrophages in the regulation of MuSC fate to ensure effective human muscle regeneration after a severe injury.

## 3 MACROPHAGES AND SKELETAL MUSCLE HYPERTROPHY

Adult skeletal muscle has a remarkable capacity to adapt to increased mechanical loading/contractile activity by increasing its mass and/or myofiber size, a phenomenon commonly refers to as skeletal muscle hypertrophy. Hypertrophy occurs when the rate of protein synthesis exceeds the rate of protein degradation and efforts have been made to decipher the cellular and molecular events occurring within the myofiber and resulting in such increased proteostasis. However, evidence is emerging that efficient muscle hypertrophy is also due to an increased number of myonuclei (i.e., myonuclear accretion) triggered by the fusion of MuSCs with the overloaded myofibers (Egner et al., 2016; McCarthy et al., 2011; Goh & Millay, 2017; Fukuda et al., 2019). So far, the role of macrophages in regulating skeletal muscle mass, possibly through interactions with the myofiber and/or other cell types such as MuSCs, is still poorly understood. Indeed, most of the existing studies have focused their interest on the kinetics of inflammatory cell accumulation in the muscle after a single session of EIMD leading to muscle damage, thus triggering muscle regeneration as described above. On the contrary, the impact of resistance training programs (i.e., multiple bouts of exercise) on the inflammatory process has been scarcely investigated as compared with the plethora of studies focusing on muscle regeneration. Considering that muscle hypertrophy could occur in the absence of substantial muscle injury (Fukada et al., 2022; Fukuda et al., 2019), there is a need for improving our understanding of how and to what extent macrophages are involved in the regulation of skeletal muscle mass.

In the following section, the role of macrophages on skeletal muscle hypertrophy will be first addressed on the basis of animal experiments. Then, human studies describing the effects of resistance training on muscle mass and macrophages will be presented. We chose to present only the results obtained from healthy muscle so experiments involving atrophic (e.g., unloading/reloading) or diseased muscle will not be considered.

### 3.1 Animal studies

The most common animal model to study hypertrophy in rodents consists in the surgical ablation of synergistic hindlimb muscles, usually *gastrocnemius* and *soleus* muscles, leaving only the remaining *plantaris* muscle to perform the daily functional activities (Armstrong et al., 1979). Such compensatory hypertrophic model (i.e., synergist ablation‐induced muscle hypertrophy) increases the mechanical loading (i.e., overload) on the *plantaris* muscle and can lead to a two‐fold increase in muscle mass and protein content according to two phases (Armstrong et al., 1979; DiPasquale et al., 2007; Marino et al., 2008). The early changes in muscle mass occurring with the first 3 days post‐overload are mainly related to an increase in muscle water content due to edema (Armstrong et al., 1979). Then, an increase in myofiber cross‐sectional area (CSA), illustrating skeletal muscle hypertrophy, usually takes places within 14 days of overload (Kandarian & White, 1989; Marino et al., 2008). Interestingly, these two phases are associated with leukocyte invasion in the muscle interstitium (Armstrong et al., 1979). The number of macrophages progressively increases, reaching a peak at day 5–7 (DiPasquale et al., 2007; Marino et al., 2008; Novak et al., 2009) and is either going back to control values (DiPasquale et al., 2007; Novak et al., 2009) or being still present (Marino et al., 2008) at day 14. These discrepancies could be related to an inter‐laboratory variability in the management of the mouse model of synergist ablation. In addition, immunohistochemical analysis of macrophage‐specific antigens indicates that both pro‐inflammatory (ED1^pos^) and anti‐inflammatory (ED2^pos^) subpopulations of macrophages increase during muscle hypertrophy, at least in aged rats (Thompson et al., 2006). Overall, synergist ablation‐induced muscle hypertrophy is characterized by a sequential infiltration then disappearance of macrophages.

Growing evidence illustrates the potential role of macrophages in hypertrophy. The first evidence was provided by DiPasquale and colleagues (DiPasquale et al., 2007) who showed that the magnitude of skeletal muscle hypertrophy is reduced after depletion of macrophages induced by intraperitoneal injection of clodronate liposomes. It was also demonstrated that hypertrophy is blunted and accumulation of macrophages is reduced in mice deficient in urokinase‐type plasminogen activator (uPA), which is in agreement with the role of uPA in macrophage chemotaxis (Bryer et al., 2008), ECM remodeling (Chapman et al., 1988) and the production of growth factors (Bryer et al., 2008). It has been recently showed that RhoA, a small GTPase protein produced by overloaded myofibers, leads to muscle hypertrophy mainly through MuSC fusion (Noviello et al., 2022). Overload‐induced myonuclear accretion was ascribed to macrophage recruitment favored by the expression of the chemokines CCL3/CX3CL1 and to ECM remodeling promoted by the upregulation of *Mmp9/Mmp13/Adam8* mRNA expression. Of note, clodronate liposomes‐induced depletion of macrophages blunts myofiber growth and diminishes both the number and fusion of MuSCs, illustrating the role of macrophages in the fate of MuSCs in response to muscle overload (Noviello et al., 2022). However, it is noteworthy that the above‐mentioned administration of clodronate liposomes results only in a partial reduction in macrophage content, so it remains to determine whether hypertrophy is totally prevented when all macrophages are lacking. Interestingly, mice deficient for β2‐integrin (CD18) that is required for leukocyte extravasation, display lower muscle mass and myofiber CSA as compared with WT mice after synergist ablation (Marino et al., 2008). In addition, gene expression of both *MyoD* and *Myogenin* is lower in CD18^−/−^ mice as compared with WT, as well as the proportion of proliferating MuSCs (i.e., BrdU^pos^Desmin^pos^ cells). However, these effects are not related to a reduction in the number of macrophages because CD18^−/−^ mice actually show a higher macrophage content at day 7 after muscle overload as compared with WT. It remains to determine whether the macrophage status (i.e., pro‐ vs. anti‐inflammatory) and/or the reduced number of neutrophils observed in the absence of CD18 play a role in the defective response to mechanical overload. Finally, Murach and colleagues (Murach et al., 2021) recently demonstrated that MuSCs modulate the expression of several chemokine genes (i.e., *Ccl2*, *Ccl5*, *Ccl7* and *Cxcl1*) in immune cells, FAPs and endothelial cells through extracellular vesicle‐mediated miR‐206/Wisp1 axis. This indicates a thin and dynamic regulation between myogenic and non‐myogenic cells to promote muscle hypertrophy (Figure 1b).

However, and importantly to be pointed out, the large number (i.e., around 25%–30%) of centrally nucleated myofibers (a marker of newly formed myofibers) observed 7 days post‐surgery (Marino et al., 2008) suggests that the accumulation of macrophages may be related at least in part to synergist ablation‐induced muscle injury, that would trigger regeneration. In addition, even though the use of tenotomy‐induced muscle overload could limit the extent of muscle injury (Fukuda et al., 2019; Kaneshige et al., 2022), the magnitude and the rate of muscle hypertrophy (i.e., +30% of muscle mass in 1–3 weeks post‐surgery) (Fukuda et al., 2019; Kaneshige et al., 2022; Noviello et al., 2022) largely exceeds what can be achieved in conventional resistance training programs (i.e., +5%–10% of muscle mass after 20–24 weeks of training) (Reggiani & Schiaffino, 2020). Although an increased number of macrophages was reported in the absence of histological alterations after electrically‐evoked concentric contractions (McLoughlin et al., 2003), the impact of the corresponding experimental protocols on muscle mass (that would have defined hypertrophy) was not documented. On that basis, the use of newly developed mouse models of exercise‐induced muscle hypertrophy relying on weight pulling mimicking resistance exercise in human (Zhu et al., 2021) or neuromuscular electrical stimulation‐induced myonuclear accretion (Zavoriti et al., 2021) could be particularly relevant to decipher the role of macrophages in this context.

### 3.2 Human studies

Only a few studies focused on the influence of macrophages on human skeletal muscle hypertrophy following resistance training protocols. It was demonstrated that the increased number of CD11b^pos^/CD206^pos^ macrophages *per* myofiber is correlated with changes in myofiber CSA, in MuSC number, in transcription of two growth factor genes (*HGF* and *IGF‐1*) and in the expression of ECM related genes (i.e., *MMP14*, *SERPIN1*, *SPARC*, *ELN*, *COL5A1*, *COL6A1*, *TGFβ1*, *LOX*) in human skeletal muscle after cycling exercise training (Walton et al., 2019). In agreement with animal studies, this suggests an active role of macrophages in the regulation of MuSCs and ECM to promote muscle growth. More recently, macrophage (i.e., CD11^pos^CD206^pos^) abundance increases in older adults submitted to 14 weeks of progressive resistance training (PRT) and is positively correlated to changes in muscle fiber size, capillary density, MuSC abundance and number of myonuclei (Peck et al., 2022), the latter suggesting MuSC fusion. Strikingly, matrix metalloproteinase 14 (*Mmp14*) gene is one of the most upregulated genes following PRT and is strongly associated with macrophage number (Peck et al., 2022), indicating that macrophages might be the main contributor of MMP14 secretion. The authors also showed in vitro that mouse macrophages treated with conditioned medium of electrically stimulated myotubes increase the expression of leukemia inhibitory factor (*Lif*) and *Mmp14* and increase collagen degradation (Peck et al., 2022). These data strongly implicate a potential role of macrophages in ECM remodeling and collagen homeostasis that could facilitate skeletal muscle adaptations to PRT through a muscle‐cell macrophage axis involving LIF and MMP14. Multiple linear regression analyses also revealed that, before PRT, collagen content/organization and macrophage abundance (CD11^pos^CD206^neg^) were trending to be associated with poor muscle growth potential (Long et al., 2022). These findings suggest that both collagen organization and macrophage content influence overall muscle growth.

In the same way, healthy individuals undergoing a 4‐week‐heavy resistance exercise training show an increased content of macrophages in the muscle endomysium near the myotendinous junction (Jakobsen et al., 2017). This elevation is accompanied by increased levels of tenascin‐C and collagen content, indicating matrix remodeling, yet it was not shown whether the exercise training protocol induces changes in myofiber CSA (Jakobsen et al., 2017). Two recent studies demonstrated that low‐load resistance training performed under blood flow restriction leads to robust muscle hypertrophy (i.e., 20%–40% increase in myofiber CSA) associated with an increase in the number of MuSCs and in the number of myonuclei *per* myofiber (Bjørnsen et al., 2019; Nielsen et al., 2012). Although the number of macrophages slightly increases during the first week of training, when the protocol leads to muscle injury (indirectly assessed by increased creatine kinase [CK] levels) (Bjørnsen et al., 2021), macrophage accumulation mainly occurs 3 weeks after the beginning of the training protocol (Bjørnsen et al., 2021; Nielsen et al., 2017). In agreement with their role in supporting muscle regeneration, macrophages could be required for the last step of myogenesis (e.g., fusion) or for interaction with other cell types such as endothelial cells or fibroblasts to promote angiogenesis and/or ECM remodeling after resistance training programs. Finally, considering that *CCL2* gene expression is increased after repeated bouts of exercise or during skeletal muscle regeneration following injury (Contreras‐Shannon et al., 2007; Hubal et al., 2008; Shireman et al., 2007), genetic variations occurring in *CCL2* and its receptor CCR2 were investigated in relation to resistance training‐induced changes in skeletal muscle size and strength (Harmon et al., 2010). In individuals undergoing a 12‐week resistance‐training program, one variant (i.e., rs1024610) in *CCL2* is associated with an increase in maximal voluntary strength, while no variant is associated with changes in skeletal muscle size in response to this training program (Harmon et al., 2010).

Overall, macrophages are mobilized into the skeletal muscle in response to resistance training programs in humans, even though the molecular regulators involved in muscle hypertrophy are still to be determined.

## 4 COMPARATIVE ANALYSIS OF THE ROLE OF MACROPHAGES IN MUSCLE REGENERATION VERSUS HYPERTROPHY

Muscle injury leads to myofiber necrosis (at least in animal models) that triggers a massive infiltration of both neutrophils and macrophages at the damaged site. On the contrary, necrosis/death of myofibers may be minimized/prevented in response to muscle overload (Fukuda et al., 2019; Kaneshige et al., 2022), resulting in a lower infiltration of macrophages and absence of efferocytosis. The inflammatory status of macrophages in response to muscle overload remains to be determined. Indeed, a clear delineation between pro‐inflammatory and restorative macrophages has been recently questioned in response to resistance exercise in humans (Jensen et al., 2020). However, the use of CD68, CD11b and CD206 markers does not allow to classify macrophage phenotype into pro‐inflammatory and restorative macrophages. Finally, while after a damage, myofibers release their internal content, triggering the inflammatory response, overloaded myofibers secrete various factors such as IL‐6 (Guerci et al., 2012; Serrano et al., 2008), IL‐4 (Guerci et al., 2012), RhoA (Noviello et al., 2022), that can dynamically regulate the interactions between macrophages, MuSCs and mesenchymal progenitors (Noviello et al., 2022) to promote muscle hypertrophy. Overall, the use of non‐damaging models of muscle hypertrophy would allow to improve our understanding of the role of macrophages and their interactions with neighboring cells in the regulation of muscle mass.

## 5 MANAGEMENT OF THE INFLAMMATORY RESPONSE

**FIGURE 2:**
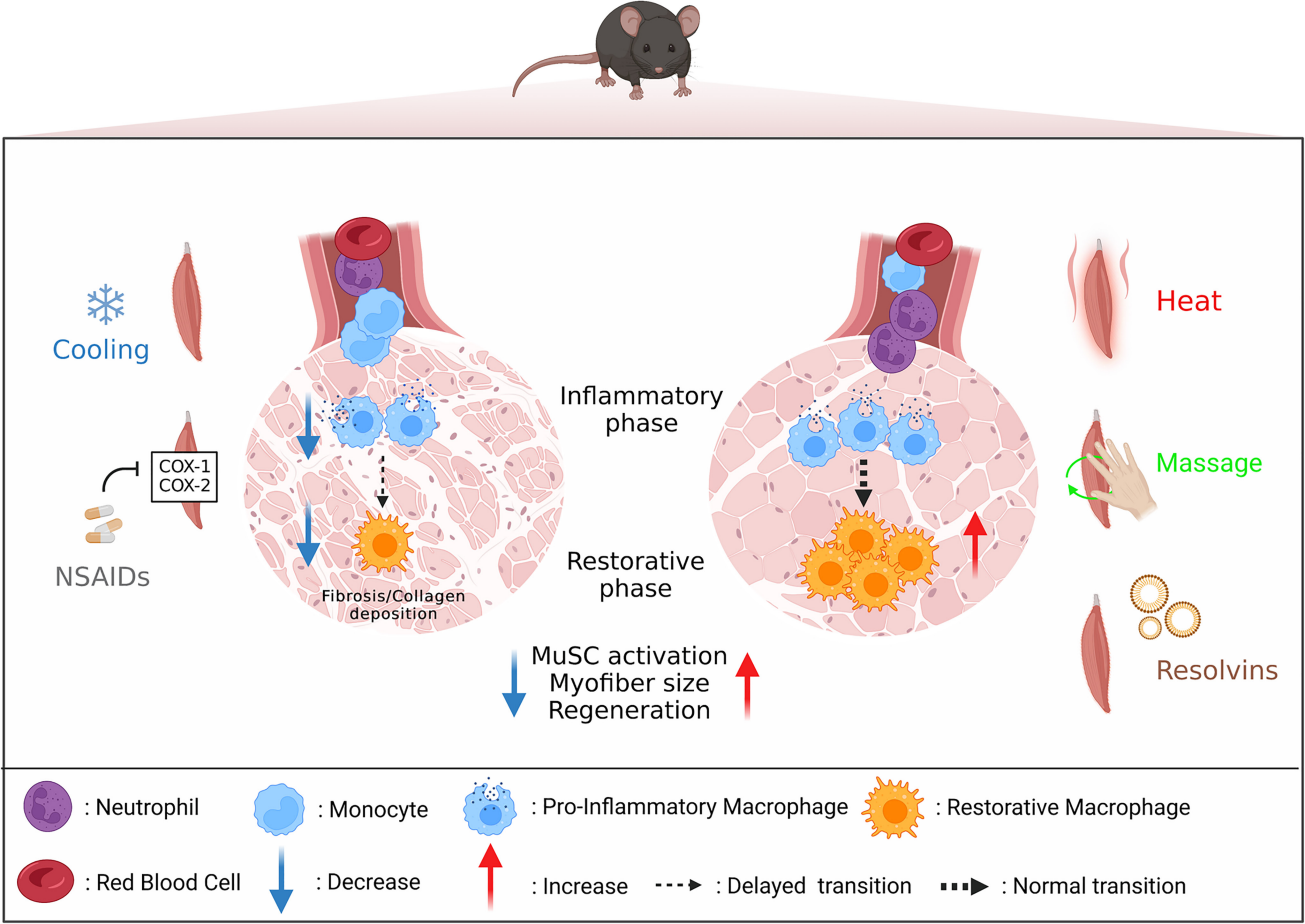
Impact of different therapeutic strategies on the muscle regeneration process in animals. Following muscle injury, cooling or administration of nonsteroidal anti‐inflammatory drugs administration (NSAIDs) can contribute to muscle regeneration defect and myofiber size decrease. Cooling decreases macrophage number and delays the shift towards anti‐inflammatory/restorative macrophages. Similarly, NSAIDs act upstream to reduce or delay macrophage infiltration through the inhibition of cyclooxygenase (COX‐1 and COX‐2) enzyme activity. As a result of perturbed inflammation, MuSCs are unable to activate properly and to repair damaged muscle fibers leading to the accumulation of necrotic muscle fibers in conjunction with collagen/fibrosis deposition. On the contrary, heating, massage or treatment with resolvins, show beneficial effects on muscle regeneration. Heating results in rapid increase of macrophages capable of resolving faster inflammation. Likewise, massage by applying cyclic movements on the injured muscle contributes to reduce the inflammatory infiltrate and favors early clearance of neutrophils. In addition, resolvins enhance the resolution of inflammation by limiting neutrophil infiltration and increasing macrophage efferocytosis. These mechanisms are associated with a better activation and differentiation of MuSCs.

Several strategies have been assessed to modulate/reduce the inflammatory response after either acute or chronic exercise training. Among them, cooling, heating, massage and nonsteroidal anti‐inflammatory drugs (NSAIDs) are widespread methods used by athletes. More recently, a class of molecules named resolvins (or specialized pro‐resolving mediators) was shown to play a key role in the resolution of inflammation in animal models of regeneration. In this section, we will primarily summarize the current knowledge on the impact of heat therapy, cooling, massage, NSAIDs and resolvins on skeletal muscle regeneration and the known underlying cellular mechanisms (Figure 2). We will also briefly discuss how these immunomodulatory strategies could affect muscle growth in response to overload. Readers can refer to excellent reviews to get a broader picture on how these therapeutic strategies impact other processes involved in skeletal muscle homeostasis (e.g., protein synthesis and degradation, adaptations to endurance/resistance training…) (Costello et al., 2015; Fennel et al., 2022; Howatson & van Someren, 2008; Hyldahl & Peake, 2020; McGorm et al., 2018; Urso, 2013; Van Pelt et al., 2021).

### 5.1 Cooling

Cooling induced by topical icing, cold‐water immersion or cryotherapy is largely used by athletes to prevent and/or treat subjective (e.g., muscle soreness) and/or objective symptoms (e.g., decrement in performance) of EIMD. However, such strategy may actually alter skeletal muscle regeneration (Shibaguchi et al., 2016; Takagi et al., 2011). Cooling decreases the number of macrophages in various models of muscle damage (Miyakawa et al., 2020; Takagi et al., 2011; Vieira Ramos et al., 2016) as well as it delays the shift towards restorative macrophages, as illustrated by a reduced number of F4/80^pos^CD206^pos^ cells, a lower IL‐10 expression and a higher TNFα expression at day 5 and day 7 post‐EIMD, respectively (Kawashima et al., 2021). Indeed, IL‐10 participates to the shift in macrophage phenotype (Deng et al., 2012) while pro‐inflammatory macrophages robustly express TNFα. In addition, cooling slows the disappearance of necrotic myofibers, suggesting an impairment of efferocytosis (Kawashima et al., 2021). The disturbed phenotypic transition of macrophages could therefore be involved in the delayed activation of MuSCs (Kawashima et al., 2021) and the increase in collagen deposition (Shibaguchi et al., 2016; Takagi et al., 2011), eventually resulting in a reduced myofiber CSA (Kawashima et al., 2021; Takagi et al., 2011).

Human studies further illustrate the lack of functional effects of cooling on muscle repair. Indeed, such strategy do neither accelerate muscle strength recovery nor reduce muscle soreness and CK activity after EIMD (Guilhem et al., 2013; Howatson et al., 2005; Tseng et al., 2013), the latter being even higher after icing as compared with a sham procedure (Tseng et al., 2013). Finally, infiltration of macrophages, changes in *Mac1* (*CD11B*, *ITGAM*), *CD163* and cytokine/chemokine mRNA expression (i.e., *IL1B*, *TNF*, *IL6*, *CCL2*, *CCL4*, *CXCL2*, *IL8* and *LIF*) are similar between cold water immersion and active recovery after a single resistance exercise. However, the moderate increase in CK activity (Peake et al., 2017) and the absence of functional measurements do not allow to ascertain the occurrence of muscle damage in this study. Finally, cold water immersion blunts myonuclear accretion, attenuates the increase in MuSC number and limits myofiber growth in response to strength training (Fyfe et al., 2019; Roberts et al., 2015). It remains however to be determined whether and to what extent these deleterious effects of cooling on muscle hypertrophy are directly due to changes in the number and/or status of macrophages. Overall, contrary to the popular belief and the usual practice, there is so far no evidence regarding the positive effects of cooling application on skeletal muscle regeneration and hypertrophy.

### 5.2 Heat therapy

Contrarily to the negative effects of cooling, the use of heat therapy following muscle injury appears as a promising therapeutic approach for promoting skeletal muscle regeneration. Various heat therapies have been used in animal studies such as hot water immersion, exposure to an environmental stress in a dedicated heat chamber or application of packs filled with hot water (Kim, Reid, et al., 2019; Kojima et al., 2007; Oishi et al., 2009; Shibaguchi et al., 2016; Takeuchi et al., 2014). Post‐injury heating treatment results in an rapid increase in the number of ED1^pos^ macrophages (Takeuchi et al., 2014), a greater number of MuSCs within the damaged muscle (Kojima et al., 2007; Oishi et al., 2009; Shibaguchi et al., 2016; Takeuchi et al., 2014) and a higher myonuclear accretion (Oishi et al., 2009) together with a faster MyoD and Myogenin protein expression (Hatade et al., 2014; Oishi et al., 2009) in response to crush or toxic injury. This results in a faster muscle regeneration illustrated by a larger myofiber CSA (Oishi et al., 2009; Shibaguchi et al., 2016; Takeuchi et al., 2014), a decrease in intramuscular collagen deposition (Shibaguchi et al., 2016; Takeuchi et al., 2014), and an improved force production after a 3‐week treatment in a mouse model of ischemia‐induced muscle injury (Kim, Reid, et al., 2019). So far, the cellular mechanisms involved in the beneficial effects of heat therapy on muscle regeneration remain unclear. Heat therapy triggers the expression of several members of the heat shock protein family (HSPs) such as *HSP25*, *HSP27*, *HSP60*, *HSP70*, *HSP72*, *HSP90* (McGorm et al., 2018) with some of them being differentially expressed in the various stages of myogenesis, suggesting specific roles for individual HSPs in this process (Thakur et al., 2019). For instance, in vitro studies showed that inhibition of HSP70 impairs myoblast differentiation (Fan et al., 2018). Interestingly, *Cd11b* and *Cd68* mRNA expression is reduced in the first 4 days post‐injury in the absence of *Hsp70* while sustained inflammation and necrosis, collagen deposition and impaired myofiber regeneration persist several weeks post‐injury in Hsp70‐deficient mice (Senf et al., 2013). In addition, heat induces the release of IL‐6 protein from myoblasts in a temperature‐dependent manner. IL‐6, which is released by myofibers in response to exercise (Pedersen & Febbraio, 2008), is known as a pleiotropic cytokine associated with the regulation of immune responses.

The substantial differences in the study design (i.e., type of exercise, heating method…) led to conflicting findings in human with studies showing either improved (Vaile et al., 2008) or unchanged (Jayaraman et al., 2004; Kuligowski et al., 1998; Pournot et al., 2011) functional performance after heating post‐EIMD. In addition, while heat therapy leads to a greater resistance to fatigue after EIMD resulting in severe functional alterations (i.e., 40%–50% reduction in voluntary strength 1 day after EIMD), it does not influence maximal voluntary strength recovery, nor macrophage content, capillarization and HSP expression (Kim, Kuang, et al., 2019). Overall, the discrepancies between animal and human studies may be related to the heating methods (whole‐body vs. localized), timing (pre, during, post‐intervention) and duration (single or repeated bouts) of interventions, underlying the need for deciphering the optimal parameters for improving or accelerating muscle regeneration. The potential benefit of heat therapy on the regulation of muscle growth has been recently highlighted (Kim et al., 2020) but the role of macrophages is still unknown.

### 5.3 Massage

Massage or mechanical/manual therapy through cyclic muscle tissue compression is a widespread method used by athletes aiming at dampening muscle soreness and/or improving recovery after a strenuous exercise (Davis et al., 2020; Howatson & van Someren, 2008). Indeed, skeletal muscle is composed of various cell types than can sense and respond to mechanical loads, a physiological process refers to as mechanotransduction (Khan & Scott, 2009). Evidence is emerging illustrating the immunomodulatory effects of massage on skeletal muscle homeostasis. In vitro experiments showed that mechanical deformation applied on a scaffold promotes the acquisition of anti‐inflammatory/restorative phenotype by macrophages (Ballotta et al., 2014). Interestingly, the application of controlled cyclic compressive loading in uninjured muscle leads to the expression of genes associated with the immune response together with the infiltration of CD68^pos^ and CD163^pos^ cells in a load‐dependent manner (Waters‐Banker et al., 2014). Several animal studies further demonstrated that four consecutive days of cyclic compression decreases the infiltration of leukocytes/macrophages and facilitates force recovery after EIMD (Butterfield et al., 2008; Haas, Butterfield, Abshire, et al., 2013; Haas, Butterfield, Zhao, et al., 2013). The effectiveness of cyclic compression on muscle regeneration is dependent on the massage timing (i.e., in favor of an immediate application as compared with a delayed intervention; Haas, Butterfield, Abshire, et al., 2013), as well as on the magnitude and frequency of mechanical loading (Haas, Butterfield, Zhao, et al., 2013). In addition, the application of a controlled massage‐like treatment after severe muscle injury resulting from notexin injection and hindlimb ischemia attenuates interstitial fibrosis, reduces inflammatory infiltrate, diminishes the proportion of pro‐inflammatory CCR7^pos^ macrophages, increases myofiber CSA and improves muscle force 14 days after injury (Cezar et al., 2016). It was suggested that in vivo tissue compression could modulate the inflammatory status of macrophages, reduce the adhesion of pro‐fibrotic macrophages and accelerate immune cell and/or metabolic waste product removal through an increased fluid transportation (Cezar et al., 2016). However, a recent study showed that cyclic muscle tissue compression‐induced functional improvement is not related to changes in macrophage content and/or inflammatory status but ascribed to an early clearance of neutrophils resulting in a reduction of the expression of several cytokines such as MMP9 and CCL3 that reduce the proliferation and/or promote the differentiation of MuSCs in vitro (Seo et al., 2021). These discrepancies are not easy to explain but could be related to different modalities of tissue compression (electromagnetic linear actuator vs. magnetic actuation of biphasic ferrogel scaffolds) and/or differences in the applied mechanical loads (Cezar et al., 2016; Seo et al., 2021). The use of precise and well‐controlled massage‐like treatment to animals (rabbits, rats and mice) (Haas, Butterfield, Abshire, et al., 2013; Hunt et al., 2019; Seo et al., 2021) should allow to improve our understanding on the impact of cyclic muscle tissue compression on the complex interaction between immune cells and neighboring cells. Human investigation also revealed that massage attenuates the production of the inflammatory cytokines TNFα and IL‐6 and mitigates the nuclear accumulation of the p65 subunit of nuclear factor kB after a cycling exercise until exhaustion (Crane et al., 2012). However, IL‐6 can be produced by other cells (e.g., myofibers; Chow et al., 2022; Guerci et al., 2012; Serrano et al., 2008) than macrophages. It is therefore unclear how and to what extent massage really improves muscle regeneration in humans through the modulation of the inflammatory response inasmuch as the investigations were also limited to the first 3 h post‐exercise. Finally, massage‐induced mechanotransduction could be beneficial for increasing muscle mass. For instance, RhoA, which controls the expression of MMPs and of macrophage chemoattractants (Noviello et al., 2022), also plays a key role in mechanotransduction by translating physical forces into biochemical signaling pathways and activating specific transcription factors. In addition, it was demonstrated that the expression of the mechanotransduction pathway Yap/Taz in mesenchymal progenitor cells of loaded muscles promotes MuSC proliferation, through a thrombospondin‐1/CD47 axis (Kaneshige et al., 2022). Massage could therefore impact macrophages and their microenvironment to promote muscle growth, even though additional studies are warranted in this context.

### 5.4 NSAIDs

NSAIDs, a class of medication reducing inflammatory processes through the inhibition of cyclooxygenase (COX‐1 and COX‐2) enzyme activity which decreases prostaglandin production from arachidonic acid, are excessively used by athletes for both prophylactic and therapeutic purposes (Gorski et al., 2011; Tscholl et al., 2015). Although a NSAID treatment administrated immediately or within 6 h (1–2 times a day for 2–7 days) after muscle injury in mice provides a short‐term functional improvement (Lapointe et al., 2002; Mishra et al., 1995; Obremsky et al., 1994), a subsequent decrement in muscle function is observed in the late phase of muscle regeneration (i.e., 28 days post‐injury) (Mishra et al., 1995). Indeed, most of the animal studies revealed that NSAIDs negatively affect muscle regeneration by reducing/delaying macrophage infiltration (Almekinders & Gilbert, 1986; Lapointe et al., 2002) and promoting fibrosis/collagen deposition via the upregulation of TGF‐β1 expression (Shen et al., 2005). The negative effects of NSAIDs are dependent of COX‐2 activity as illustrated by the altered muscle regeneration in mice deficient for COX2 (Bondesen et al., 2004; Shen et al., 2006) or treated with selective COX‐2 inhibitors (Bondesen et al., 2004; Shen et al., 2005) and the preserved muscle regeneration in mice treated with a selective COX‐1 inhibitor (Bondesen et al., 2004). This could be ascribed to the role of COX‐2 in macrophage chemotaxis (Reding et al., 2006) and in uPA activity. It was further demonstrated that prostaglandin E2 plays a key role in the proliferation of MuSCs (Ho et al., 2017) and that NSAIDs may exert a negative effect on MuSC fate in vitro (Liao et al., 2019) and in vivo (Ho et al., 2017). Overall, a consensus is emerging on the deleterious effects of NSAIDs in animal models of skeletal muscle regeneration even though it remains to be determined how they disturb cellular interactions in this context.

Contrarily to animal experiments, findings are still controversial regarding the role of NSAIDs after EIMD in humans. These discrepancies could be related, at least in part, to the magnitude of histological alterations (e.g., number of desmin‐negative myofibers) and the extent of macrophage infiltration which can be less severe in human as compared with animals (see Section 1). It has been consistently demonstrated that NSAID treatment has no effect on the number of macrophages (Mikkelsen et al., 2009; Paulsen et al., 2010; Vella et al., 2016) or on the mRNA levels of *Cd68* (Mackey et al., 2016), suggesting that the effects of NSAIDs on human muscle function are not necessarily mediated by the regulation of immune cells. Interestingly, NSAIDs could affect the fate of MuSCs after exercise, even though this is not a universal finding (Paulsen et al., 2010). Two studies reported that NSAIDs attenuate the increase of MuSC number after voluntary endurance or eccentric exercise that are not associated with overt signs of muscle injury/regeneration (e.g., absence of desmin‐negative myofibers or embryonic myosin heavy chain positive myofibers) (Mackey et al., 2007; Mikkelsen et al., 2009), illustrating a role for the COX pathway in the regulation of MuSC activity in humans. This is consistent with in vitro studies showing that prostaglandins are involved in the fusion of primary chick myoblasts (Zalin, 1977). On the contrary, a greater increase in the proportion of MuSCs was observed after administration of NSAIDs as compared with placebo 7 days after a severe muscle injury induced by electrical stimulation applied during lengthening contractions (Mackey et al., 2016). In addition, NSAIDs induce a faster muscle repair as illustrated by the return of both MuSC content and ECM gene expression to baseline values, suggesting dynamic interactions between MuSCs and fibroblastic cells while the role of macrophages is not described in this context. Overall, one could assume that NSAIDs differentially impact macrophages and their interactions with neighboring cells in damaging versus non‐damaging conditions. However, independently of the extent of injury, NSAIDs seem to fasten muscle function recovery when assessed 24–48 h post exercise (Bourgeois et al., 1999; Hasson et al., 1993) but do not result in long‐term functional improvement (Mackey et al., 2016; Paulsen et al., 2010). The contradictory findings in humans could be due to the large differences in the exercise protocols and the related severity of injury, the timing (prophylactic vs. therapeutic) and/or the duration of treatment (e.g., single dose (Hasson et al., 1993) vs. a 6‐week treatment (Mackey et al., 2016)), the number of included subjects, as well as the type of drugs (i.e., selective vs. non‐selective COX inhibitors). Finally, blunted skeletal muscle hypertrophy has also been reported after chronic administration of NSAIDs (Lilja et al., 2018; Novak et al., 2009; Soltow et al., 2006) at least in young healthy muscles. This was associated with a reduced macrophage infiltration (Novak et al., 2009) which is in agreement with the role of COX‐2 in macrophage chemotaxis (Reding et al., 2006). On the contrary, positive effects of NSAIDs on skeletal muscle mass and/or force have been described in healthy elderly adults (Trappe et al., 2011), suggesting that the efficacy of this strategy may rely on the basal inflammatory status. Further studies are needed to illustrate how macrophages are impacted by NSAIDs in response to overload in both young and older muscles.

### 5.5 Resolvins

The inflammatory response is sustained by different classes of lipid mediators: the pro‐inflammatory phase is associated with prostaglandins (which synthesis relies on COX1 and COX2) and leukotrienes. The repair/healing phase is supported by a series of more than 40 lipids with anti‐inflammatory properties including lipoxins, resolvins, maresins and protectins, called Specialized Pro‐resolving Mediators (or SPM) (Serhan, 2014). Resolvins are a family of more than 20 members formed from omega‐3 essential polyunsaturated fatty acids, docosahexaenoic acid (DHA), eicosapentaenoic acid (EPA) and docosapentaenoic acid (n‐3 DPA) giving rise to the D‐, E‐ and T‐series resolvins, respectively (Serhan, 2014). So far, five resolvin receptors have been identified (A lipoxin and formyl peptide receptor 2 [ALX/FPR2], DRV1/GPR32, DRV2/GPR18, ERV1/ChemR23, GPR101) belonging to the seven transmembrane G‐protein coupled receptor family (Cash et al., 2014; Flak et al., 2020). Resolvins actively participate in the resolution of inflammation, notably by limiting neutrophil infiltration and increasing macrophage efferocytosis.

In skeletal muscle, promising results have been recently obtained with the use of resolvins. In a hindlimb ischemia injury model, where skeletal myofibers are injured and therefore where muscle operates regeneration, a daily treatment with Resolvin D2 from 24 h after injury improves angiogenesis and increases the number of regenerating myofibers 14 days after injury, associated with a decrease in neutrophil infiltration in the muscle at early time points (Zhang et al., 2016). Treatment with Resolvin D1 leads to a similar improvement and requires signaling through its ALX/FPR2 receptor on macrophages. Indeed, in LysM^Cre^;ALX/FPR2^flox^ mice, where the gene is deleted in myeloid cells, the expression of a provascularization phenotype of macrophages in blunted (Sansbury et al., 2020). In both toxin‐induced muscle injury and EIMD, the lipid mediator class shift usually observed during the inflammatory response occurs at the time of resolution of inflammation, notably in macrophages (Giannakis et al., 2019; Markworth et al., 2020). A single intramuscular injection of Resolvin D2 in early regenerating muscle (day 2–3 after toxic injury) improves both muscle mass and force recovery, and increases the number of Ly6C^neg^ restorative macrophages (Giannakis et al., 2019). A single intraperitoneal injection of Resolvin D2 at the time of injury strongly decreases neutrophil infiltration at early time points (from day 1 post‐toxic injury) and induces the expression of the restorative phenotype of macrophages at a later stage (increased expression of *Alox12*, *Arg1*, *IL1b*) (day 5), associated with an improvement of the myogenic process (Markworth et al., 2020). In both studies, resolvin administration leads to increased myofiber CSA and muscle strength (Juban, 2021; Trappe et al., 2011). It is noteworthy that Resolvins also impact on myogenesis, at least in vitro. Resolvin D1 at 100 nM has no impact on myogenesis in the murine myoblast cell line C2C12 but counteracts the deleterious effect of high concentrations of TNFα (20 ng/ml) on myogenesis (Markworth et al., 2020). Resolvin D2 at 200 nM directly stimulates primary mouse MuSC differentiation and myogenesis, through the GPR18 receptor that is expressed in differentiating muscle stem cells (Dort et al., 2021).

Most interestingly, these studies have directly compared the impact of resolvins and other anti‐inflammatory treatments. Resolvins have a superior benefit for muscle regeneration than NSAIDs (ibuprofen), that acutely inhibits the pro‐inflammatory prostanoids, but their expression rebounds later on, driving strong unbalance of the kinetics of macrophage subtypes (the transition to the acquisition of the restorative phenotype is impaired) (Giannakis et al., 2019) and that glucocorticoids (prednisolone is less efficient and with only short‐term benefit) (Dort et al., 2021).

## 6 CONCLUSION

Macrophages are at the heart of the regeneration process by interacting with MuSCs and other cell types to restore skeletal muscle structure and function. Growing evidence is also emerging on their role on skeletal muscle hypertrophy. The use of non‐damaging mouse models of exercise should allow to determine how they participate in the regulation of myofiber size. This should clearly highlight the specific role of macrophages and their interactions with neighboring cells in muscle regeneration versus hypertrophy. Animal studies highlight the key role of several immunomodulatory strategies to favor muscle regeneration and evidence is emerging illustrating that the modulation of the inflammatory response could be relevant to favor muscle hypertrophy. Rather than blunting the pro‐inflammatory phase, promoting the active resolution of inflammation to establish the regenerative inflammation appears a good strategy to improve/accelerate muscle regeneration as well as to promote muscle growth.

## AUTHOR CONTRIBUTIONS

Clara Bernard, Aliki Zavoriti, Quentin Pucelle, Bénédicte Chazaud and Julien Gondin contributed to the conception and design of this review. Clara Bernard, Aliki Zavoriti, Quentin Pucelle, Bénédicte Chazaud and Julien Gondin interpreted and synthesized the data. Clara Bernard, Aliki Zavoriti, Quentin Pucelle, Bénédicte Chazaud and Julien Gondin wrote the draft manuscript. All authors contributed to and revised the final manuscript. All authors read and approved the final manuscript.

## Supporting information


Figure S1
