# Supplementary figures and images for: Role of macrophages during skeletal muscle regeneration and hypertrophy—Implications for immunomodulatory strategies
Source: Physiol Rep. 2022 Oct 6;10(19):e15480. doi: 10.14814/phy2.15480 (PMC9535344; doi:10.14814/phy2.15480)

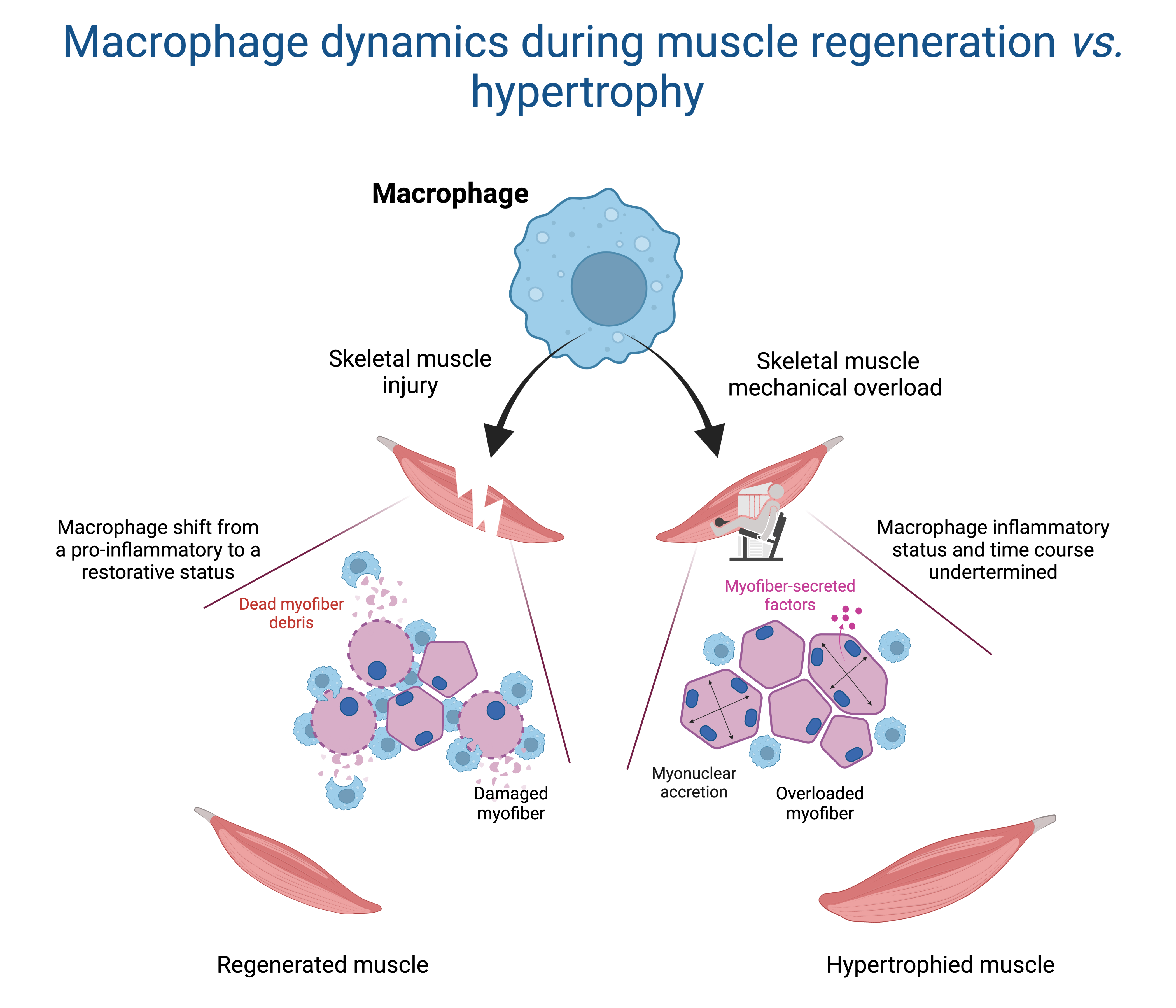

Supplement: Supplementary file 1 — Figure S1 [file PHY2-10-e15480-s001.png]
